# Improving TDOA Radar Performance in Jammed Areas through Neural Network-Based Signal Processing

**DOI:** 10.3390/s23062889

**Published:** 2023-03-07

**Authors:** Jakub Gotthans, Tomas Gotthans, David Novak

**Affiliations:** 1 Department of Radio Electronics, Brno University of Technology, 61600 Brno, Czech Republic; 2 Department of Communication Technologies, Electronic Warfare and Radiolocation, University of Defense, 66210 Brno, Czech Republic

**Keywords:** TDOA, radar, autoencoder, neural network, deep neural network, jamming, correlation method

## Abstract

This paper presents a method for estimating the position of a target under jammed conditions using the Time Difference of Arrival (TDOA) method. The algorithm utilizes a deep neural network to overcome the challenges posed by the jammed conditions. The simulations and results indicate that the presented method is more accurate and efficient than the traditional TDOA methods.

## 1. Introduction

Time Difference of Arrival (TDOA) is a technique used to determine the location of a transmitter by measuring the time difference of arrival (TDOA) of a signal at multiple receivers. TDOA location estimation is based on the principle that the signal from the transmitter will arrive at each receiver at a slightly different time due to the distance between the transmitter and the receivers. By measuring the time difference of arrival of the signal at each receiver, it is possible to determine the transmitter’s location. TDOA location estimation can be performed using either a time-based or frequency-based approach. In a time-based approach, the time difference of arrival is directly measured by comparing the signal arrival times at each receiver. This can be done using high-precision clocks to measure the arrival time of the signal at each receiver. In a frequency-based approach, the frequency difference of arrival is measured and used to calculate the time difference of arrival. This can be done by measuring the frequency shift of the signal due to the Doppler effect caused by the relative motion between the transmitter and the receivers. TDOA location estimation can also be performed using either a single-tone or a multi-tone approach. In a single-tone approach, a single frequency is used for the transmitted signal, while in a multi-tone approach, multiple frequencies are used. The use of multiple frequencies allows for the use of frequency-based TDOA estimation, which can be more accurate than time-based TDOA estimation in certain situations.

One of the challenges in TDOA location estimation is the need to accurately synchronize the clocks of the receivers, as even small clock errors can significantly impact the accuracy of the TDOA measurements. To mitigate the impact of clock errors, TDOA location estimation can be performed using a network of receivers rather than a single receiver, which allows for calculating clock error compensation values.

Another challenge in TDOA location estimation is the presence of multi-path interference, which occurs when the transmitted signal takes multiple paths to reach the receivers due to reflections off of nearby objects. This can cause errors in the TDOA measurements and degrade the accuracy of the location estimation. Multi-path interference can be mitigated through the use of advanced signal processing techniques such as matched filtering and least squares estimation [1].

TDOA location estimation can also be affected by the presence of noise and interference in the environment [2], which can degrade the signal-to-noise ratio (SNR) of the received signal. To improve the SNR and, thus, the accuracy of TDOA location estimation, advanced techniques such as adaptive filtering and Kalman filtering can be used.

A key challenge with contemporary radar systems is their requirement for high spatial resolution, which necessitates the use of wide-band sampling. However, the transfer of sampled signals to a central processing node can be an arduous and demanding task. While optical links can be utilized to aggregate several receiving nodes, such an approach is often impractical. To address this issue, this paper presents a novel method that employs a deep neural network to compress the sampled signal into a latent space, which can then be transferred to a central processing node for data processing, where the signal is expanded and analyzed.

Furthermore, the incorporation of convolution layers in the neural network enables the system to be robust against jamming. This approach is explored in the subsequent sections of this paper and evaluated using the constructed simulator.

### 1.1. State-of-the-Art

Overall, TDOA location estimation is a powerful tool for determining the location of a transmitter in a wireless communication system, with applications in a wide range of fields, including military and defense, transportation, and emergency response. Ongoing research in TDOA location estimation aims to improve accuracy, reliability, and efficiency, as well as to explore new applications and integration with other technologies.

TDOA location estimation can be traced back to the early 20th century with the development of the hyperbolic positioning system by Marconi and Braun in 1903. In the 1950s, the development of radar systems led to the use of TDOA for target tracking and location estimation [3]. In the 1970s, the advent of cellular communication systems led to the development of TDOA-based location estimation techniques for mobile phone systems. In the 1980s, the Global Positioning System (GPS) was developed, which used a combination of TDOA and angle of arrival (AOA) measurements [4] for satellite-based location estimation [5]. In the 1990s, the widespread adoption of wireless networking technologies such as Wi-Fi and Bluetooth led to the development of TDOA-based location estimation techniques for these systems. In the early 21st century, the Internet of Things (IoT) and the increasing demand for location-based services led to further research and development in TDOA location estimation techniques. Some works already combined neural networks with TDOA systems, combining the outputs of all the individual NN to improve position estimate accuracy [6].

Two techniques for three-dimensional target localization using bistatic range readings from several transmitter-receiver pairs in a passive radar system were first introduced by Malanowski and K. Kulp in 2012. The algorithms, called spherical interpolation (SI) and spherical intersection (SX), are based on methods used in TDOA systems and use closed-form equations. The paper includes a theoretical accuracy analysis of the algorithms, verified through Monte-Carlo simulations and a real-life example [7].

In 2015 A. Noroozi and M. A. Sebt presented a closed-form weighted least squares method for determining the position of a target in a passive radar system with multiple transmitters and receivers using TDOA measurements. The method involves intersecting ellipsoids defined by bistatic range (BR) measurements from various transmitters and receivers. The localization formula is derived from minimizing the weighted equation error energy. To improve the method’s performance, the paper proposes two weighting matrices, one leading to an approximate maximum likelihood (ML) estimator and the other to a best linear unbiased estimator (BLUE). The paper includes numerical simulations to support the theoretical developments [8].

An improved approach for localizing a moving target utilizing a noncoherent multiple-input multiple-output (MIMO) radar system with widely dispersed antennas was presented in 2016 by H. Yang and J. Chun. The method is based on the two-stage weighted least squares (2SWLS) approach but only requires a single reference transmitter or receiver and can easily incorporate time-of-arrival (TOA), frequency-of-arrival (FOA), TDOA, and frequency-difference-of-arrival (FDOA) data. The authors also introduce auxiliary variables to improve numerical stability and demonstrate that their method is more stable than the Group-2SWLS method while achieving the Cramer–Rao lower bound (CRLB) at higher noise levels [9].

In 2017 A. Noroozi and M. A. Sebt presented a method for estimating the location of a single target using bistatic range measurements in a multistatic passive radar system. The proposed method uses a weighted least squares (WLS) approach to eliminate nuisance parameters, which are parameters that are unknown and cannot be estimated from the data, and to obtain an estimate of the target location. The method involves several WLS minimizations, and two different weighting matrices are derived to improve the method’s performance. One of these matrices leads to the maximum likelihood estimator (MLE), while the other leads to the best linear unbiased estimator (BLUE) [10].

In 2020 F. Ma, F. Guo, and L. Yang addressed the problem of directly determining the positions of moving sources using the received signals. Traditional methods for localization of moving sources involve two steps. To identify the location of the moving sources, estimations of the intermediate TDOA and FDOA characteristics are performed. In contrast, the authors propose a new method that directly estimates the locations and velocities of moving sources from the received signals. To solve the problem of simultaneously estimating the high-dimensional unknown parameters, the authors propose a multiple particle filter-based method, in which the positions and velocities of the moving sources are updated alternately using separate local particle filters. The proposed method requires fewer particles compared to classic particle filters, and its convergence is proved both theoretically and numerically. The Cramer–Rao lower bound for the proposed moving source localization method is also developed. Simulation results show that the proposed method is computationally efficient and accurate in estimating the positions of moving sources [11].

A method for calculating the location characteristics of a moving aerial target in an Internet of Vehicles (IoV) system employing space-air-ground-integrated networks was proposed by Liu et al. in 2022. (SAGINs) [12]. The proposed method uses multiple satellites to estimate the TDOA and FDOA signals received from the moving aerial target. The distance between the target and the receiver, as well as the velocity of the moving aerial target, are then estimated using the TDOA and FDOA estimates. To suppress direct-path and multipath interference in the received signals, the authors first filter the direct wave signals in the reference channels using a band-pass filter and then apply a sequence cancellation algorithm. The time and frequency differences of arrival are then estimated using the fourth-order cyclic cumulant cross ambiguity function (FOCCCAF) of the signals in the reference channels and the four-weighted fractional Fourier transform FOCCCAF (FWFRFT-FOCCCAF) of the signals in the surveillance channels. The Cramer–Rao lower bounds of the proposed location parameter estimators are also derived to benchmark the performance of the estimators. Simulation results show that the proposed method can effectively and accurately estimate the location parameters of the moving aerial target [12].

In recent years, advances in signal processing algorithms and machine learning techniques have led to improved accuracy and efficiency in TDOA location estimation. TDOA location estimation has also been integrated with other location estimation techniques, such as angle of arrival (AOA) and signal strength (RSS) measurements, to improve accuracy and reliability [13,14,15].

TDOA location estimation is now used in a wide range of applications, including military and defense, transportation, emergency response, and the development of advanced technologies such as autonomous vehicles and smart cities. In the future, TDOA location estimation is expected to play a key role in developing intelligent transportation systems and advancing communication technologies such as 5G and beyond. Ongoing research in TDOA location estimation aims to improve accuracy, reliability, and efficiency, as well as to explore new applications and integration with other technologies. Some of the current areas of research in TDOA location estimation include the development of advanced signal processing algorithms, the use of machine learning techniques, and the integration of TDOA with other location estimation techniques such as AOA and RSS measurements.

**Figure 1:**
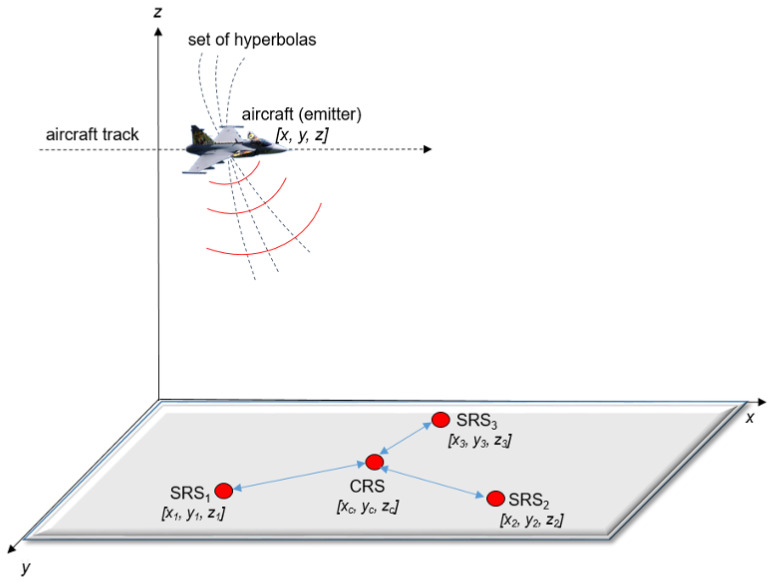
General configuration of the PET TDOA system.

Passive electronic support measurement tracker (PET) systems are also known as passive surveillance systems (PSS), and their principles and technology are highly similar to radar technology from a passive radar point of view [16]. Many PET systems are based on the multilateration TDOA method to determine the exact position of targets and can ordinarily track them. The target for this article means an emitter, which is placed on a moving platform and emits electromagnetic signals such as radar, radios, etc. PET TDOA systems are mostly dedicated to land, air, sea, and space situational awareness (SA) in military or non-military purposes such as air traffic control (ATC) and other uses [17,18]. In the case of SA ATC, the PET TDOA system utilizes a secondary surveillance radar aircraft transponder’s reply signals to locate, identify, and track aircraft in the large area [17]. If a 3D target position is required, a PET TDOA consists of four receiving stations, such as the central receiving station (CRS) and three side receiving stations (SRS1, SRS2, SRS3), see Figure 1.

All receiving stations are placed in terrain with radio visibility towards the target, and the exact coordinates $\left(x_{i},y_{i},z_{i}\right)$ of CRS, SRS1, SRS2, and SRS3 are known. All signals emitting from the target received by CRS, SRS1, SRS2, and SRS3 are led to the signal processing where the TDOA method is applied [17,18]. The TDOA method relies on the knowledge of the receiver’s coordinates and the time of signal arrival at each receiving station to compute the hyperbolic coordinates of the emitter. CRS is considered a reference. The general configuration of the four-position PET TDOA system is shown in Figure 1. This TDOA measurement between CRS and SRS1 establishes an isodelay (hyperbolic) curve that passes through the emitter (target) location. The second hyperbola is calculated between CRS and SRS2, and the last is established between CRS and SRS3. The intersection of the set of hyperbolas locates the emitter, as shown in Figure 1.

### 1.2. Neural Networks

A neural network is a type of artificial intelligence system that is inspired by the structure and function of the human brain. An input layer, one or more hidden layers, and an output layer are only a few of the layers of interconnected neurons or nodes that make up this system. Numerous tasks, including as classification, regression, and function approximation, are carried out using neural networks. They are particularly well-suited for tasks where the input and output are fixed-length, and the relationships between them are well-defined [19].

Neural networks are trained using an optimization algorithm, such as stochastic gradient descent (SGD), which adjusts the weights and biases of the network to minimize the error between the predicted output and the true output. This process is known as backpropagation, which involves propagating the error back through the network and updating the weights and biases in each layer [19].

The capacity of neural networks to learn from and adjust to incoming data is one of its main advantages. They can learn complex relationships between the input and output data and generalize to new data, making them effective at tasks such as image classification and speech recognition [19].

Despite their success, neural networks do have some limitations. They can be sensitive to the quality and scale of the input data and may require preprocessing or data augmentation to achieve good results. In addition, neural networks can be computationally expensive to train, requiring specialized hardware and optimization techniques to achieve good performance [19].

Feed-forward neural networks, recurrent neural networks, and convolutional neural networks are a few of the several types of neural networks. Each type is designed for specific types of tasks and data, and selecting the appropriate type of neural network for a given task is an important consideration in the design and implementation of an AI system [19].

In this research, we suggest combining several neural networks. Since neural networks are nowadays penetrating all fields of science, we wondered if they could have a benefit in TDOA systems. Therefore, in the following paragraphs, we describe in general terms the different neural networks we have used in the design of the network architecture [19].

#### 1.2.1. Perceptron

One layer of neurons or nodes makes up a perceptron, an artificial neural network. Frank Rosenblatt developed it in the 1950s as a way to simulate the learning process of the human brain. Perceptrons are used for various tasks, including classification, regression, and function approximation. They are particularly well-suited for tasks where the input and output are fixed-length, and the relationships between them are well-defined [19].

The structure of a perceptron consists of an input layer and an output layer, with the input layer receiving the raw input data and the output layer producing the final prediction or output. The input layer is connected to the output layer through weights and biases, which are adjusted during the training process to minimize the error between the predicted output and the true output [20].

One of the key advantages of perceptrons is their simplicity and ease of implementation. They are relatively easy to train and can be implemented using a variety of programming languages and libraries. In addition, perceptrons can be highly efficient, especially with hardware acceleration, such as graphics processing units (GPUs). In addition, perceptrons are limited in their ability to model complex relationships between the input and output data. They are only able to learn linear decision boundaries, making them less effective at tasks that require more complex decision boundaries [20].

**Figure 2:**
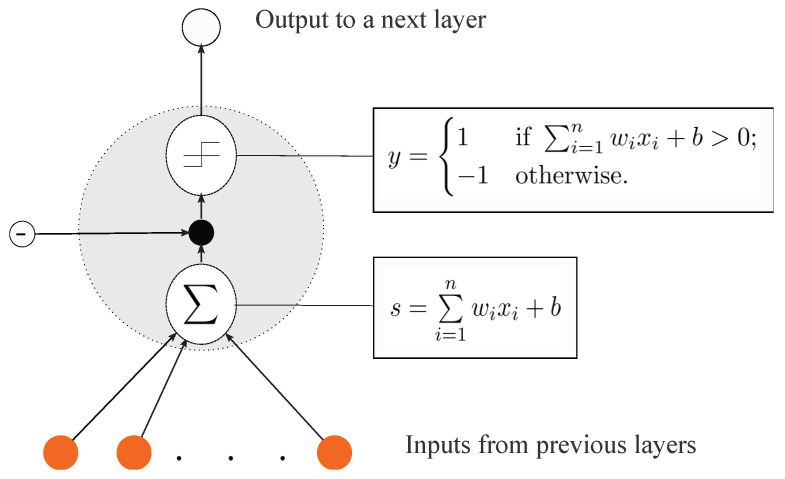
General structure of the perceptron [20].

Given the input vector $x=\langle x_{1},\ldots ,x_{n}\rangle$ and trained weights $W_{1},\ldots ,W_{n}$, the perceptron output *y* is represented by formula [20]:

(1) $$y=\left\{\begin{array}{cc} 1, & \mathrm{if}\;\sum_{i=1}^{n}W_{i}x_{i}+b>1;\\ -1, & \mathrm{otherwise}, \end{array}\right.$$

where $\sum_{i=1}^{n}W_{i}x_{i}$ is the weighted input and s=z+b is the state of the perceptron. For the perceptron to be activated, the threshold value must exceed its state *s* [20]. The Boolean operations AND, OR, NAND, and NOR are just a few that the perceptron may express. Figure 2 depicts the perceptron’s general structure [20].

#### 1.2.2. Feed Forward Networks

**Figure 3:**
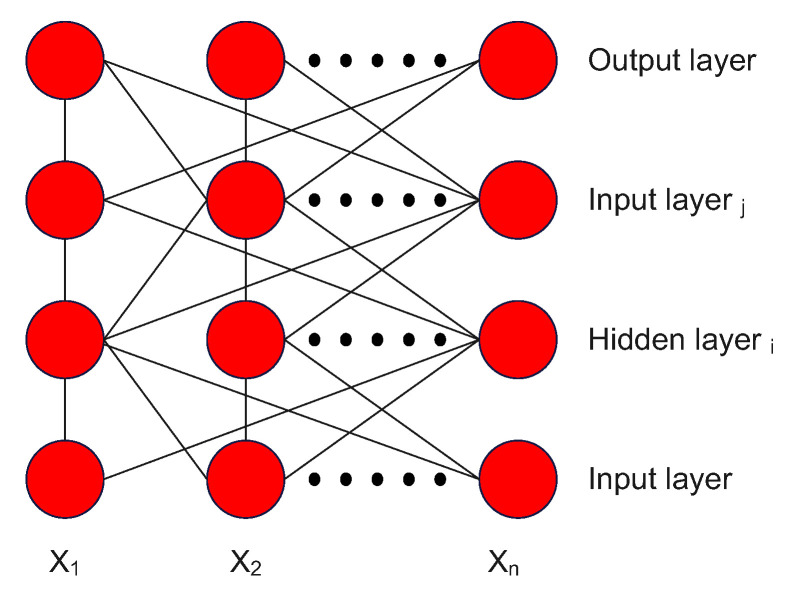
Multilayer feed-forward network with an input layer, two hidden layers, and an output layer [20].

A feed-forward neural network, also known as a feed-forward network or a feed-forward artificial neural network, is a type of artificial neural network that consists of layers of interconnected neurons or nodes. It is called "feed-forward" because the information flows through the network in one direction, from the input layer to the output layer, without looping back. Feed-forward networks are used for various tasks, including classification, regression, and function approximation. They are particularly well-suited for tasks where the input and output are fixed-length, and the relationships between them are well-defined. The number of layers and neurons in each layer determines the structure of a feed-forward network. The input layer receives the raw input data, while the output layer produces the final prediction or output. The layers in between are called hidden layers, and the neurons in these layers are responsible for learning and extracting features from the input data. The main functionality of the feed-forward network is to approximate some function $f^{*}$. For example, we have classifier $y=f^{*}\left(x\right)$ map an input *x* to a category *y*. A feed-forward network defines a mapping y=f(x;θ) and learns the value of the parameters θ that result in the best function approximation [21]. The structure of layers and connections is shown in Figure 3.

These models are called feed-forward because information flows through the function evaluated from *x*, through the intermediate computations used to define *f*, and finally to the output *y*. There are no feedback connections in which outputs of the model are fed back into itself [21].

A group of neurons represents the feed-forward neural network. Each of these layers of neurons compute a weighted sum of its inputs. Depending on the neuron’s location in the network, we can distinguish the basic levels of neurons. The first level is the input neurons, through which environmental signals are emitted. A group of neurons represents the feed-forward neural network. Each of these layers of neurons compute a weighted sum of its inputs. The next type is output neurons, which pass processed signals back to the environment. The last type is hidden neurons, which are inside the network and do not interact with the external environment. Feed-forward neural networks have no loops and are completely integrated. This indicates that no weights provide input to a neuron in the previous layer and that every neuron from the previous layer is connected to every neuron in the subsequent layer. Bias values are used to initialize the weights of a feed-forward neural network to small, normalized random numbers. The neural network is then trained using all training samples, and error backpropagation computes each unit’s input and output for all (hidden and visible)output layers [20].

Feed-forward networks are trained using an optimization algorithm, such as SGD, which adjusts the weights and biases of the network to minimize the error between the predicted output and the true output. This process is known as backpropagation, which involves propagating the error back through the network and updating the weights and biases in each layer [20].

One of the key advantages of feed-forward networks is their simplicity and ease of implementation. They are relatively easy to train and can be implemented using a variety of programming languages and libraries. In addition, feed-forward networks can be highly efficient, especially with hardware acceleration such as GPUs. Despite their simplicity, feedforward networks can be powerful tools for solving many problems. They have been used to achieve state-of-the-art performance on tasks such as image classification and speech recognition [20].

However, feed-forward networks do have some limitations. They are not well-suited for tasks that require incorporating context or dependencies over time, such as natural language processing or speech recognition. For these tasks, recurrent neural networks (RNNs) or convolutional neural networks (CNNs) are typically used [20].

Feed-forward neural networks are a widely used and powerful tool for solving various tasks in machine learning and artificial intelligence. They are an active area of research and will likely continue to be an important tool in developing intelligent systems.

#### 1.2.3. Convolutional Neural Networks

Artificial neural networks known as CNNs are particularly effective at recognizing objects in images and videos. They are called “convolutional” because they use a mathematical operation called convolution to analyze the input data. This operation allows the network to learn features and patterns in the data by sliding a small matrix, called a kernel or filter, over the input and performing element-wise multiplications and summaries.

**Figure 4:**
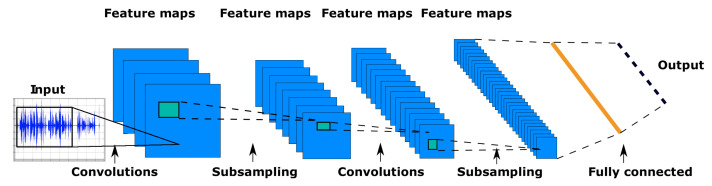
Typical CNN network.

CNNs are composed of several layers of neurons, each responsible for learning a different aspect of the input data. The first layers of a CNN typically learn simple features, such as edges and corners, while the deeper layers learn more complex features, such as shapes and patterns. The output of the final layer is a prediction of the class or label of the input data. The typical architecture of CNN is shown in Figure 4 [22].

One of the key advantages of CNNs is their ability to learn features directly from the input data rather than requiring manual feature engineering. This allows them to achieve high performance on tasks such as image classification and object detection. In addition, CNNs can use their learned features to generalize to new data, making them effective for tasks such as image synthesis and style transfer [22].

Despite their success, CNNs do have some limitations. They can be sensitive to the quality and scale of the input data and may require preprocessing or data augmentation to achieve good performance. In addition, CNNs can be computationally expensive to train, requiring specialized hardware and optimization techniques to achieve good performance [22].

CNNs are widely utilized in applications including computer vision, natural language processing, and speech recognition, because they have consistently shown to be effective tools for image and video recognition tasks [22].

CNNs are feed-forward neural networks with modified architecture. The architecture of CNNs usually consists of convolutional layers followed by a pooling layer, where each neuron in a convolutional layer is connected to some region in the input. This region is usually called a local receptive field. All weights (filters) in CNNs are shared based on the position within a receptive field. The convolution operation can be described as follows [22]:

(2) $$\left(f*g\right)\left(z\right)=\sum_{x}\sum_{y}f\left(x,y\right)\cdot g\left(z-x,z-y\right),$$

where f(x,y) is the input image at position (x,y) and g(z−x,z−y) is a trainable filter. The pooling layers in CNN reduce the dimensionality of features, which leads to a reduction of connection between the layers. Hence it reduces the computational time [23]. In our particular case, we used autoencoders that consist of convolutional layers. The autoencoders aim to reduce a large amount of data from individual antenna nodes.

#### 1.2.4. Recurrent Neural Network

**Figure 5:**
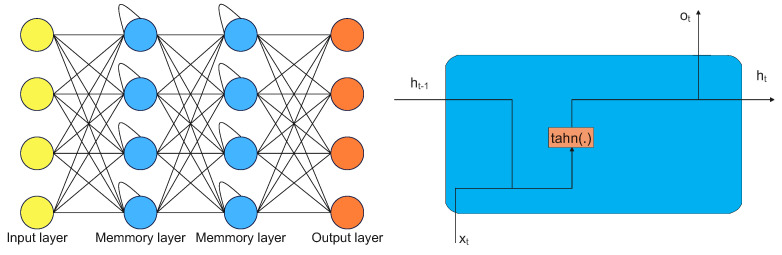
Typical RNN mechanism.

RNNs are a form of artificial neural network that excel at handling sequential input, including time series, speech, and spoken language. They are called “recurrent" because they use feedback connections, allowing the network to retain information from previous time steps and use it to inform its current output. This makes RNNs capable of learning long-term dependencies and patterns in sequential data. A typical example of RNN architecture is shown in Figure 5 [21].

Several RNNs, including the long short-term memory (LSTM) network and the gated recurrent unit (GRU) network are used. These architectures introduce special "memory" cells and gating mechanisms that allow the network to selectively retain and forget information as needed, helping to prevent the vanishing and exploding gradient problems that can occur in traditional RNNs. RNNs can be used for various tasks, including language translation, language modeling, machine translation, and text generation. They are also used in speech recognition, music generation, and robot controlds [21,24].

One of the key challenges in training RNNs is the need to process the entire input data sequence simultaneously, which can be computationally expensive. To address this issue, techniques such as truncated backpropagation through time (BPTT) and teacher forcing can reduce the sequence length that needs to be processed [21].

Despite their success, RNNs do have some limitations. They can be difficult to train, especially for longer sequences, and may require careful optimization and regularization techniques to achieve good performance. In addition, RNNs can be sensitive to the quality and scale of the input data and may require preprocessing or data augmentation to achieve good results [21].

One of the key advantages of RNNs is their ability to incorporate context from previous time steps into their predictions. This makes them particularly well-suited for tasks such as language translation, where the meaning of a word can depend on the words that come before and after it. Another advantage of RNNs is their ability to handle variable-length sequences. This makes them useful for tasks such as machine translation, where the length of the input and output sequences can vary greatly [21,24].

One of the most successful applications of RNNs is natural language processing (NLP). RNNs have been used to achieve state-of-the-art performance on tasks such as language translation, language modeling, and sentiment analysis. In addition to their success in NLP, RNNs have also been used to achieve good results in speech recognition tasks. They have been used to model the temporal dependencies in speech signals, allowing them to learn the important patterns and features for recognizing different sounds and words [21].

Overall, recurrent neural networks are a powerful tool for processing sequential data and have found wide application in natural language processing and speech recognition tasks. Despite their challenges, they have proven to be a valuable tool for solving a wide range of problems and are an active area of research in machine learning and artificial intelligence [21].

Typical recurrent memory architecture is shown in Figure 5, where $h_{t},C_{t}$ represent hidden layer vectors, $X_{t}$ is the input vector, $b_{h}$ is the bias vector, $\sigma_{h},\sigma_{y}$ are the activation functions, and U,W,V are the parameter matrices. All relations are described as follows:

(3) $$\begin{array}{c} h_{t}=\sigma_{h}\left(i_{t}\right)=\sigma_{h}\left(U_{h}x_{t}+V_{h}h_{t-1}=b_{h}\right),\\ y_{t}=\sigma_{y}\left(a_{t}\right)=\sigma \left(W_{y}h_{t}+b_{h}\right). \end{array}$$

#### 1.2.5. Long-Short Term Memory

LSTM is a type of RNN that is particularly well-suited for processing sequential data with long-term dependencies. Hochreiter and Schmidhuber introduced it in 1997 to solve the vanishing gradient problem that affects traditional RNNs. LSTMs are composed of special “memory” cells that can retain information for extended periods and gating mechanisms that allow the network to retain or forget information as needed selectively. The input, forget, and output gates allow the LSTM to control the flow of information into and out of the memory cells, while the cell state serves as the internal memory of the LSTM [21].

LSTMs have been used to achieve state-of-the-art performance on tasks such as language translation, language modeling, machine translation, and speech recognition. They have proven to be particularly effective at capturing long-term dependencies in sequential data, allowing them to learn complex patterns and structures. One of the key advantages of LSTMs is their ability to handle variable-length sequences, making them useful for tasks such as machine translation, where the length of the input and output sequences can vary greatly. They can also incorporate context from previous time steps into their predictions, making them well-suited for tasks such as language translation, where the meaning of a word can depend on the words that come before and after it [25].

Despite their success, LSTMs do have some limitations. They can be computationally expensive to train and require careful optimization and regularization techniques to achieve good performance. In addition, LSTMs can be sensitive to the quality and scale of the input data and may require preprocessing or data augmentation to achieve good results [25].

One area of active research in the field of LSTMs is the development of more efficient architectures and training techniques. This includes using techniques such as weight tying and pruning to reduce the number of parameters in the network, as well as using optimization algorithms such as Adam and SGD with momentum to speed up training [25].

Another area of research is the development of LSTM variants that are better suited for specific tasks or types of data. For example, attention mechanisms have been introduced to allow LSTMs to focus on specific parts of the input sequence when making predictions [25].

Overall, LSTMs have proven to be a powerful tool for processing sequential data and have found wide application in natural language processing and speech recognition tasks. They are an active area of research in machine learning and artificial intelligence. They will likely continue to be an important tool in developing intelligent systems [25].

Each memory block in the original architecture contained an input and output gate. The input gate controls the flow of input activations into the memory cell. The output gate controls the output flow of cell activations into the rest of the network. Later, the forget gate was added to the memory block [25].

**Figure 6:**
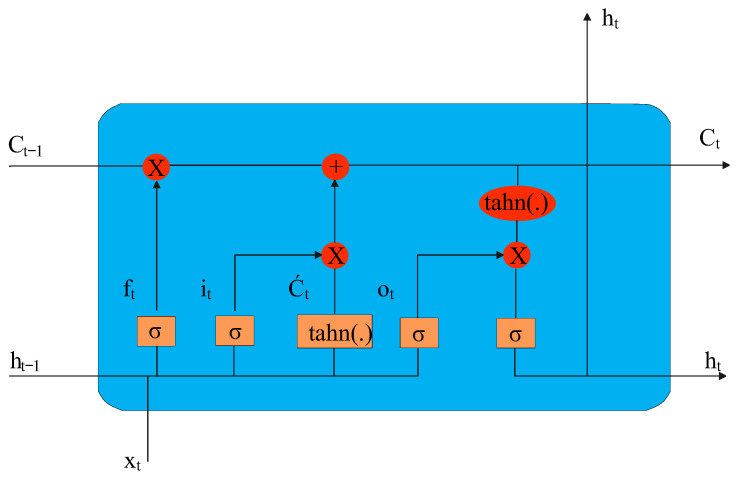
Typical LSTM network [26].

A typical LSTM architecture is shown in Figure 6, where $h_{t},C_{t}$ represents the hidden layer vectors, $X_{t}$ is the input vector, $b_{i},b_{c},b_{f},b_{o}$ are the bias vectors, and σ, tanh are the activation functions. Functions $f_{t},i_{t},\tilde{C}$ are described as follows:

(4) $$\begin{array}{c} f_{t}=\sigma \left(W_{f}\left[h_{t-1},x_{t}\right]+b_{f}\right),\\ i_{t}=\sigma \left(W_{i}\left[h_{t-1},x_{t}\right]+b_{i}\right),\\ o_{t}=\sigma \left(W_{o}\left[h_{t-1},x_{t}\right]+b_{o}\right),\\ {\tilde{C}}_{t}=\left(W_{c}\left[h_{t-1},x_{t}\right]+b_{c}\right),\\ C_{t}=f_{t}⊙C_{t-1}+i_{t}⊙{\tilde{C}}_{t},\\ h_{t}=o_{t}⊙\tanh\left(C_{t}\right). \end{array}$$

#### 1.2.6. Gated Recurrent Unit

A particular kind of RNN that excels at processing sequential data with long-term dependencies is the gated recurrent unit (GRU). It was introduced by Cho et al. in 2014 as a simplified version of the LSTM network, which was developed to address the vanishing gradient problem that affects traditional RNNs. GRUs are composed of special "memory" cells and gating mechanisms that allow the network to selectively retain or forget information as needed. The update and reset gates control the flow of information into and out of the memory cells, while the cell state serves as the internal memory of the GRU [25].

Micro-Doppler Effect and Determination of Rotor Blades by Deep Neural Networks Speech recognition, language modeling, machine translation, and other activities have all benefited from the employment of GRUs. They have proven particularly effective at capturing long-term dependencies in sequential data, allowing them to learn complex patterns and structures. One of the key advantages of GRUs is their simplicity and efficiency compared to LSTMs. They have fewer parameters and require less computation, making them faster to train and easier to optimize. They can also handle variable-length sequences, making them useful for tasks such as machine translation, where the length of the input and output sequences can vary greatly [25].

Despite their success, GRUs does have some limitations. They may not be as powerful as LSTMs on certain tasks, especially those that require more complex memory mechanisms. In addition, GRUs can be sensitive to the quality and scale of the input data and may require preprocessing or data augmentation to achieve good results [25].

One area of active research in the field of GRUs is the development of more efficient training techniques. This includes using techniques such as weight tying and pruning to reduce the number of parameters in the network, as well as using optimization algorithms such as Adam and SGD with momentum to speed up training. Another area of research is the development of GRU variants better suited for specific tasks or data types. For example, attention mechanisms have been introduced to allow GRUs to focus on specific parts of the input sequence when making predictions [25].

GRUs have proven to be a useful tool for processing sequential data and have found wide application in natural language processing and speech recognition tasks. They are an active area of research in machine learning and artificial intelligence and will likely continue to be an important tool in developing intelligent systems [25].

**Figure 7:**
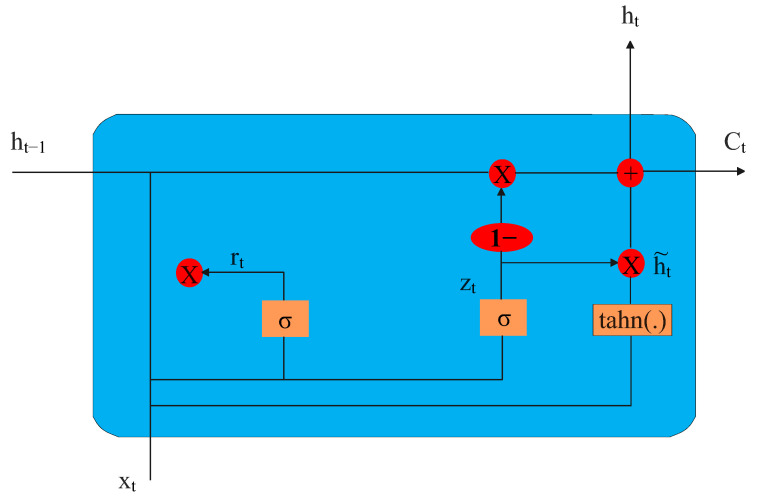
Typical GRU unit architecture [26].

The typical LSTM architecture is shown in Figure 7, where $h_{t}$ represents hidden layer vectors, $X_{t}$ is the input vector, $b_{z},b_{r},b_{h}$ are the bias vectors, $W_{z},W_{r},W_{h}$ are the parameter matrices, and σ, tanh are the activation functions. Functions $f_{t},i_{t},\tilde{C}$ are described as follows:

(5) $$\begin{array}{c} z_{t}=\sigma \left(W_{z}\left[h_{t-1},x_{t}\right]+b_{z}\right),\\ r_{t}=\sigma \left(W_{r}\left[h_{t-1},x_{t}\right]+b_{r}\right),\\ {\tilde{h}}_{t}=\left(W_{h}\left[r_{t}⊙h_{t-1},x_{t}\right]+b_{h}\right),\\ h_{t}=\left(1-z_{t}\right)⊙h_{t-1}+z_{t}⊙\begin{array}{c} h_{t} \end{array} \end{array}$$

### 1.3. TDOA Detection Methods

TDOA is a technique used to determine the location of a radio transmitter based on the difference in the time it takes a radio signal to reach different receivers. It works by measuring the time difference between when a signal is received at two or more different locations and then using that information to triangulate the transmitter’s position. TDOA is based on the principle that the speed of light *c* is constant and that the time it takes for a radio signal to travel from the transmitter to each receiver can be accurately measured. This method can be used for both passive and active location systems, and it is commonly used in military and civilian applications such as wireless communication, navigation, and surveillance.

There are several advantages to using the TDOA method:

- It requires only a single antenna per sensor and at least four sensors for 3D location estimation;
- TDOA can provide higher precision and accuracy compared to other location estimation methods.

However, there are also some disadvantages to using TDOA:

- Accurate and synchronized clocks are required for each sensor to ensure the accuracy of the TDOA estimation;
- TDOA estimation accuracy can be affected by measurement errors on sensor positions, the multipath problem (signal reflection), the sensors’ timing accuracy, and the sensors’ geometry concerning the target.

**Figure 8:**
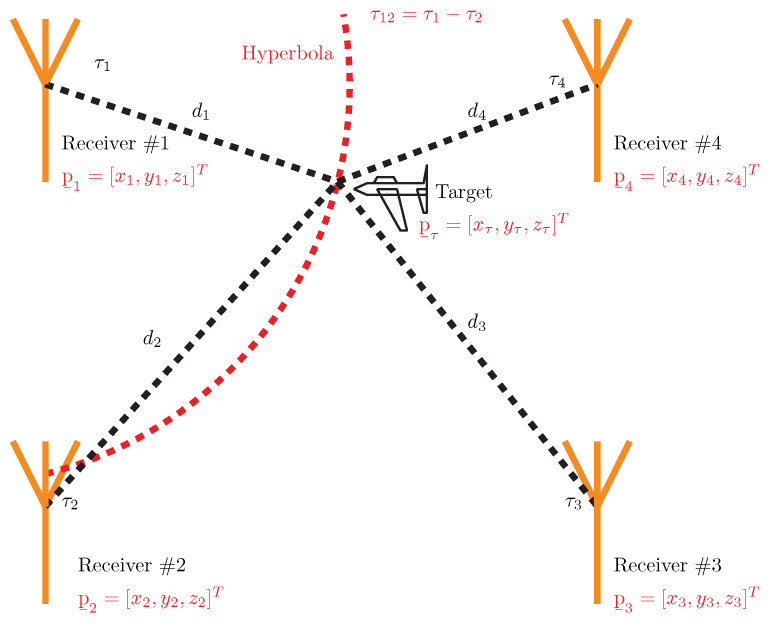
Typical TDOA scenario.

In a TDOA scenario, it is assumed that the sensors are stationary and synchronized, resulting in no Doppler shifts for any of the sensors. To estimate the TDOA, the cross-correlation between signals received by different sensors is typically calculated using a classical approach. TDOA localization scenario is shown in Figure 8. Each sensor receives the signal with some delay in time and frequency. The received signal for *i*-th sensor is shown as,

(6) $$y_{i}\left(t\right)=e^{j\alpha_{i}}e^{-jw_{di}t}s\left(t-\tau_{i}\right)+n\left(t\right),i=1,2,...,M$$

where $\alpha_{i}$ is the phase introduced by the time of flight of the signal and $w_{di}$ is the Doppler frequency shift of *i*-th sensor with velocity where $\alpha_{i}=w_{c}\tau_{i}$ and $w_{di}=\frac{w_{c}v_{i}}{c}$. Hence, the TDOA value between the first and second sensors can be determined by locating the peak of the cross-correlation function $|R_{y_{1}y_{2}}\left(\tau_{12}\right)|$

(7) $$R_{y_{1}y_{2}}\left(\tau_{12}\right)=\int_{0}^{T}y_{1}\left(t\right)y_{2}^{*}\left(t-\tau_{12}\right)dt.$$

Finding the peak of $|R_{y_{1}y_{2}}\left(\tau_{12}\right)|$ gives a TDOA value between 1st and 2nd receiving antenna $\tau_{12}=\tau_{2}-\tau_{1}$, we can write $\tau_{12}$ by using the distance $d_{1}$ and $d_{2}$ as $\tau_{12}=\frac{d_{2}-d_{1}}{c}$ and using propagation speed (speed of light $c=310^{8}\;m/s$. Some works have used unknown propagation speed, such as in Ref. [27]) *c* as $d_{2}=d_{1}+c\tau_{12}$. To generalize, we can estimate the distances for points given by Cartesian coordinates in n-dimensional Euclidean space between the antenna and target *T* as the Euclidean distance:

(8) $$d_{i}=\left|\right|p_{i}-p_{T}\left|\right|.$$

The equation can be rewritten for clarity and emphasis as follows (using the Cartesian coordinates x,y,z:

(9) $$d_{i}^{2}={\left(d_{1}+c\tau_{1i}\right)}^{2}={\left(x_{i}-x_{T}\right)}^{2}+{\left(y_{i}-y_{T}\right)}^{2}+{\left(z_{i}-z_{T}\right)}^{2},i=2,3,\ldots M.$$

The equation can be solved, e.g., by Taylor series expansion (using at least four sensors) or by adding a new variable and doing the linearization (at least five receiving antennas are needed). Note that time differences are not affected by errors in the receiver’s clock time as it cancels out when subtracting two measurements.

For simplicity, the task is reduced to 2D, and the position is calculated as follows [28]:

(10) $$H=\left[\begin{array}{cc} x_{2} & y_{2}\\ x_{3} & y_{3}\\ x_{4} & y_{4} \end{array}\right],C=\left[\begin{array}{c} -d_{21}\\ -d_{31}\\ -d_{41} \end{array}\right],D=\left[\begin{array}{c} P_{2}^{2}-d_{21}^{2}\\ P_{3}^{2}-d_{31}^{2}\\ P_{4}^{2}-d_{41}^{2} \end{array}\right],$$

which yields the following least-squares intermediate solution:

(11) $$\hat{x}={\left(H^{T}H\right)}^{-1}H^{T}\left(d_{1}C+D\right)$$

where $P_{i}^{2}=x_{i}^{2}+y_{i}^{2}$.

## 2. Proposed Architecture

A system simulator was developed to assess the principle, which was written entirely in Python language. The source code for the simulator can be found on GitLab [29]. The simulator is used to conduct unsupervised training and evaluation of neural networks. To test the system’s jamming resistance, a jammer based on Zadoff–Chu sequences was constructed. These sequences are ideal candidates due to their correlation properties that are capable of confusing radar detectors. The subsequent subsection describes the subsystems that were utilized.

### 2.1. Dataset

In the radars, the BPSK signals in the form of the Barker codes are widely used. In this paper, we have used the quadrature phase shift keying (QPSK) modulation, which has been widely applied in communication systems. The QPSK-modulated signals have a low error rate, strong anti-jamming ability, and low complexity. We assume that the QPSK signal is reflected from the target. The receiver carrier frequency is $f_{c}=10\;\mathrm{GHz}$, and the sampling frequency is 1GSps.

### 2.2. Jamming

Zadoff–Chu (ZC) sequences are complex sequences with unit amplitude and specific phase shifts and are now widely used in modern cellular systems such as LTE and 5G NR. They have replaced previous spread spectrum sequences, such as PN and Walsh sequences, commonly used in 3G cellular systems (WCDMA and CDMA2000) and IS-95. Unlike Walsh and PN codes, which are real and binary-valued (usually 1±), ZC sequences have a different structure. ZC sequences have several remarkable and desirable properties, such as the cyclic auto-correlation of a ZC sequence being optimal. Hence such sequences can be used as jammers to overload the receivers. The simulation generates a PN (Pseudo-Noise) sequence using a Linear Feedback Shift Register (LFSR) for jamming purposes. A Zadoff–Chu sequence has two key parameters, (i) the root index $q=1,2,\ldots ,N_{zc}-1$ and (ii) the length of the sequence, $N_{zc}$, which must be an odd number (and is often a prime number). Given these two parameters, the qth ZC sequence $s_{q}\left[n\right]$ is defined as:

(12) $$s_{q}\left[n\right]=exp\left(-j\pi q\frac{n(n+1)}{N_{zc}}\right),$$

where $n=0,1,2,\ldots ,N_{zc}-1$. Note that each sequence has length $N_{zc}$, while the number of such sequences is $N_{zc}-1$. The jamming signals are added with 50% probability.

### 2.3. Test Setup

For testing purposes, a TDOA simulator was created in Python. The main idea is to create a simple region representing the field of view. Four antennas are placed at each corner point, and our goal is to detect a moving object inside the evaluated area. Our simulator generates a reflected signal in the moving object, and then according to the distance from each antenna node, the signal is delayed and attenuated. The signals are concentrated in the central node. To reduce data floating from the antenna to the central node, only one of the quadrature components (I or Q) has been used. Then the differential time delays are calculated. The operation of the simulator was verified by correlating the signals from individual antenna nodes and calculating the object’s position.

The simulator being discussed is a system that simulates the behavior of a radio signal received by multiple antennas. It consists of four receiving antennas that are distributed in space. The purpose of this simulator is to simulate the movement of a target in space and how the radio signal reflected off of the target is affected by the environment as the antennas receive it.

One of the key features of this simulator is the ability to account for the varying delays that occur as the antennas receive the signal. Various factors, such as the distance between the target and the antennas, can cause these delays. The simulator can extract these delays by cross-correlations from the signals received by the antennas and use them to calculate the target’s position.

If the goal is to confuse a radar system, one way to achieve this is by using a jamming device, which is a device that transmits signals specifically designed to disrupt or interfere with the operation of the radar. In our case, the jammer is a generator of ZC sequences, which are complex sequences with unit amplitude and specific phase shifts. These sequences can overload and confuse the correlation detector of the radar system, making it impossible to detect the target reliably. This is known as jamming the radar.

In our simulator, we placed the jammers randomly in the environment to make it very difficult for the radar system to mitigate the jamming. This added realism to the simulation, mimicking the unpredictable nature of jamming in real-world scenarios. The second task of our TDOA simulator was to generate an interference signal that would preclude the use of standard detection methods based on signal correlation. This was done to simulate the impact of jamming on the radar system and to test the effectiveness of different jamming countermeasures. In the simulation, the receiving antenna positions are #1 antenna [0, 0] m, #2 antenna [1000, 0] m, #3 antenna [0, 1000] m, and #4 antenna [800, 700] m. The carrier frequency is $f_{c}=10\;\mathrm{GHz}$, and the sampling frequency is 1GSps. The signal from the target to the receiver is attenuated according to the:

(13) $$A_{dB}=20\;log_{10}\left(distance\right)+20\;log_{10}\left(f_{c}\right)-147.55.$$

**Figure 9:**
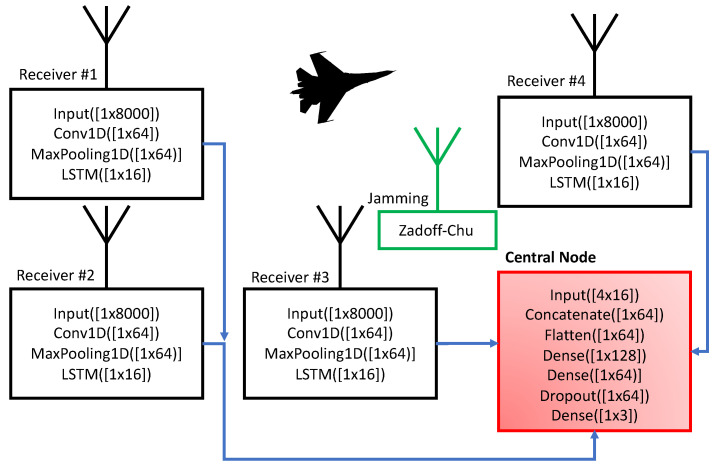
Description of the test-bed. The simulator has four receiving antennas, each connected to a neural network. Then the signals (with major dimension reduction) are concentrated in the central node. The central node is used for delay estimation.

The block diagram describing the principle can be seen in Figure 9. The simulator has four fixed receiving antennas, each connected to a neural network. Then the signals (with major dimension reduction) are concentrated in the central node. In principle, the receiver-central node neural networks work as auto-encoder. The central node is used for delay estimation.

**Figure 10:**
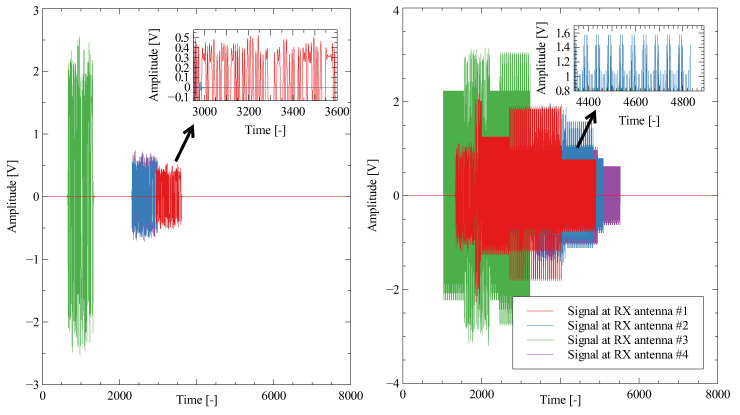
Example of the received signals. There is only one component from the quadrature receiver on four receiving antennas. **Left**: Signals without jamming. **Right**: Signals with randomly placed jamming devices.

**Figure 11:**
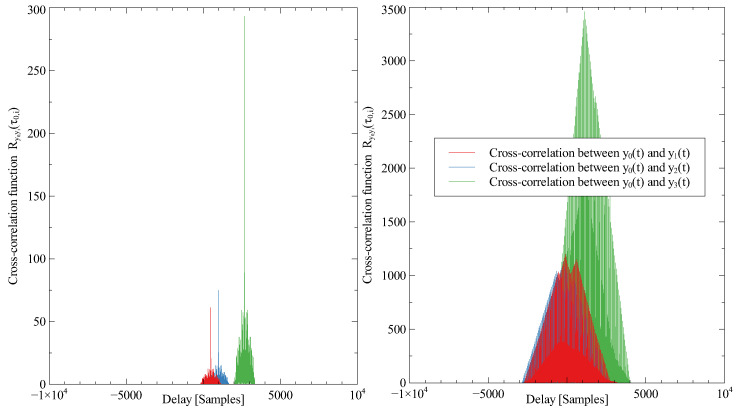
Example of the cross-correlation function for delay estimation. **Left**: Signals without jamming. **Right**: Signals with the randomly placed jamming device. If the jammer is used, the signals reflected from the target are hidden. Hence the algorithm is not able to estimate the correct delays.

Signals received by antennas can be seen in Figure 10. There is only one component from the quadrature receiver on four receiving antennas. Signals without jamming can be easily used for delay estimation by cross-correlation. However, if the signals are intercepted with strong jamming signals (randomly distributed in space), the generic method cannot extract the delay. The neural network can compress the sparse information to reduced state space. By using convolution layers and memory layers such as LSTM or GRU, the network can filter out unwanted signals and provide relatively satisfactory results. An example of a cross-correlation function for delay estimation with and without jamming signals can be seen in Figure 11.

### 2.4. Deep Neural Network Architecture—RadarNET

**Figure 12:**
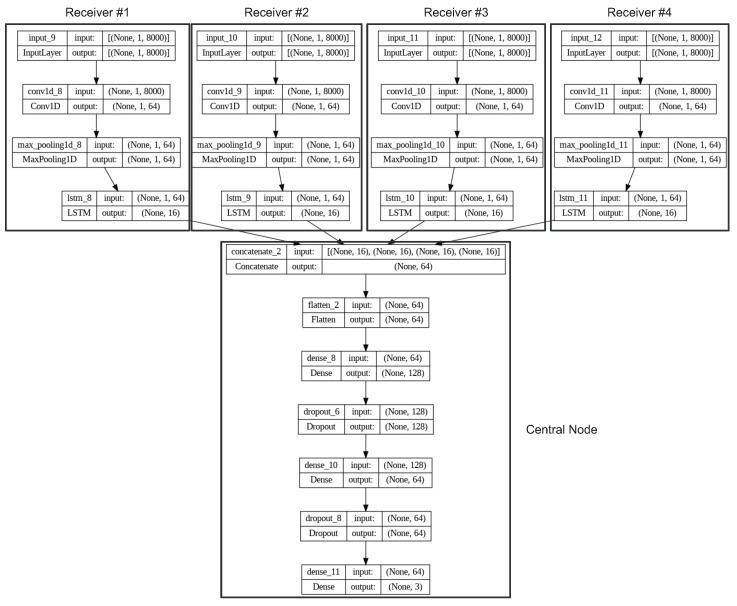
RadarNET: a neural network employed for filtration, compression, and delay estimation that utilizes a unique structure. The input node is duplicated and distributed to all four receiving antennas, ensuring that each antenna node has the same network structure and coefficient values. This allows for efficient and consistent processing of incoming signals, ensuring accurate and reliable results.

The neural network is used for two specific tasks. First, it is used to compress the signal from the receiving antenna to the central node. Secondly, it is used for resistance against jamming. For this purpose, the authors have designed RadarNET, as described below. At the input of each antenna node, there is a buffer of 8000 samples, and the output is encoded only to 16 samples. To provide the capability to be scaled, the input node is cloned (all four nodes have the same network structure and same coefficients) to four receiving antennas. The real input signal is injected into the 1D convolution layer, followed by the Max-pooling layer to provide filtration of the artifacts. Then the signal is processed by a LSTM. This series of layers works as a filter—finding specific patterns related exclusively with the reflected radar signals. Then the LSTM cell encodes the filtered signal in to a latent subspace made of 16 real-valued samples representing the features of the reflected signal. The cell remembers values over arbitrary time intervals and compresses the signal further to 16 samples. In general, the input part of the network can be seen as a autoencoder. Another important idea is incorporated in the image, which is to clone coefficients to other input units, the reason being to make on-site implementation possible (implementation directly to a receiving antenna unit). Hence, each antenna node provides 16 samples, that is, 64 samples in total, from 4 receiving antennas to the central node. The structure of the neural network can be seen in Figure 9 and Figure 12.

The central node collects compressed data from the antenna nodes, then increases the vector size from 64 to 128 samples. This part can be seen as a autodecoder network structure scaling the latent space to 12 samples. The layer is followed by fully connected layers providing a time-difference estimation outputting three samples that represent the time differences. The differences are used in Equation (11) to calculate the position of the target. To ensure accurate calculations, a Dropout layer with a probability of 0.2 is employed. The network then outputs mutual delays. The neural network boasts an impressive architecture consisting of 10,277,763 trainable parameters. This large number of parameters allows for a high degree of flexibility and complexity in the network’s decision-making processes, enabling it to process and analyze the input data effectively. The network can also continuously improve its performance through training, utilizing this vast number of parameters to learn and adapt to new patterns and structures within the data.

To overcome the challenges posed by a large number of coefficients and memory limitations, the data was loaded and processed on demand. To guarantee the convergence of both the evaluation and training datasets, a dynamic loading strategy was implemented. The data for training was generated anew for each evolution, comprising 200 batches for training and 100 batches for evaluation. The training process employed the Mean Squared Error (MSE) criterion, utilizing the ADAM optimizer within the Keras framework for optimal performance. This approach not only allows for the efficient utilization of computational resources but also ensures that the network’s performance continually improves over time as it processes new and diverse data.

## 3. Results

To evaluate the proposed algorithm, using neural networks, the simulator for receiving reflected signals from a moving target was created. The scripts written in Python language can be found on GitLab [29]. In the simulator, there are four receiving antennas measuring the reflected signals at a carrier frequency of 1 GHz with a sampling frequency of 1 GSps. The simulations were tested on 1000 measurements of data (which is sufficient for average calculation) and compared with a classical algorithm [7]. Two scenarios were considered—with one jammer and with two jammers. The performance of the algorithms was evaluated by calculating the total error as the average and maximum errors. The results of the comparisons are shown in Table 1 and Table 2, and it can be observed that the new algorithm using neural networks outperforms the classical approach under jamming conditions. The new algorithm achieved a lower average and maximum error in both scenarios, demonstrating its improved performance in the presence of jamming. This research shows that the proposed algorithm is a promising solution for addressing jamming challenges in wireless communication systems.

**Figure 13:**
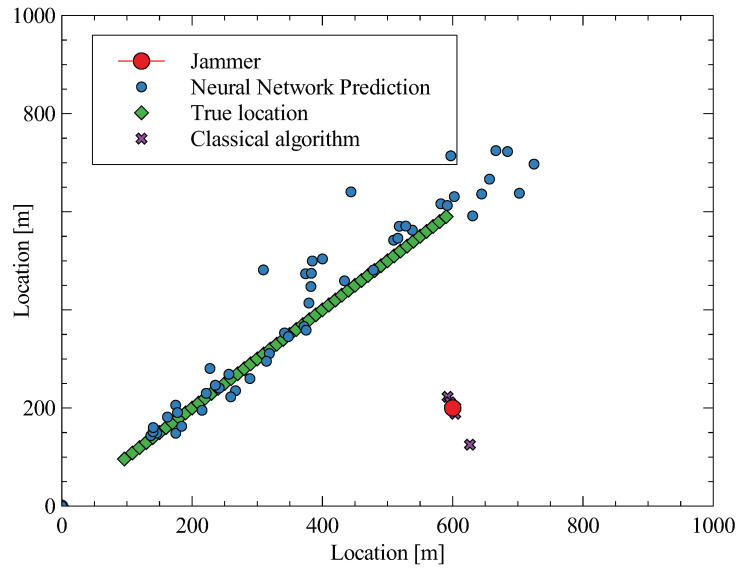
Linear trajectory of the target- green was generated. The jammer was located at [600, 200] m. The neural network predictions are blue. The classical algorithm [7] is violet. It demonstrated confusion about the estimation of the target position. The classical algorithm was evaluated with similar quantities as the neural network approach. However, some violet crosses overlap due to a limited number of cross-correlations.

The proposed algorithm was evaluated on a linear trajectory of the target to provide meaningful results. The jammer was strategically located at the position [600, 200] m. The evaluation results were visually represented in the form of plots in Figure 13, with the neural network predictions illustrated in blue dots and the predictions of the classical algorithm illustrated in violet. The plots demonstrate the superior performance of the proposed algorithm, as it accurately detects the target, while the classical algorithm exhibits confusion in its estimation of the target’s position. This highlights the effectiveness of the proposed algorithm in addressing the challenges of jamming in TDOA systems. The classical algorithm was evaluated with similar quantities as the neural network approach. However, some violet crosses overlap due to a limited number of cross-correlations.

## 4. Discussion

In this paper, a method for estimating the position of a target under jammed conditions using the Time Difference of Arrival method is presented. Additionally, a significant reduction of data transmission between the antenna nodes and the central node is achieved through advanced signal processing techniques. The algorithm utilizes a deep neural network to overcome the challenges posed by the jammed conditions. The neural network employed boasts a highly sophisticated architecture featuring a total of 10,277,763 trainable parameters. Each antenna node has a buffer of 8000 samples at its input, and the output is compressed to only 16 samples. Despite the compression, the network still demonstrates superior performance compared to traditional methods, such as cross-correlation for delay estimation. This approach not only allows for the efficient utilization of computational resources but also ensures that the network’s performance continually improves over time as it processes new and diverse data.

Furthermore, the network is designed to be robust against noise and interference, ensuring accurate and reliable results even in challenging jammed environments. The results of the simulations show that the proposed method outperforms the conventional TDOA method in terms of accuracy and computational efficiency. The use of deep neural networks in this application opens up new possibilities for developing advanced signal-processing algorithms for positioning systems and other related fields. The proposed method can be applied to various scenarios, such as indoor and outdoor localization, navigation, and tracking of moving targets.

The receiver antennas are distributed in the square perimeter and for convenience, the target is only moving inside. However it is possible to be outside the perimeter. The reader may observe significant maximum position error of the so-called classical method [7]. The classical method uses cross-correlation, as described by Equation (7), of the received signals in order to estimate mutual delays. With a sampling rate of 1 GSps and a signal length of 8000 samples, the maximum cross-correlation offset can be anticipated to reach up to 4000 samples. Considering the speed of light as the propagation speed for the electromagnetic signal, this offset corresponds to a maximum mutual distance of approximately 12,000 meters. Hence, in the worst-case scenario, the maximum possible error in the simulator can reach 16 km.

## 5. Conclusions

In this paper, the authors have presented the concept of using a neural network to compress the sampled signal into a latent space, which can then be transferred to a central processing node for data processing, where the signal is expanded and analyzed.

Furthermore, the incorporation of convolution layers in the neural network enables the system to be robust against jamming. The network is designed to have an antenna compressing (processing) node with a neural network independent in the field. The compressed signals from several nodes are aggregated in the main node and are analyzed.

Many papers, such as Refs. [6,13,30], have presented the use of neural networks as a complement to standard methods to pinpoint the location of an object. However, the authors believe that so far no work has considered the use of jammed signals and the usage of neural networks as an autoencoder for radars, which reduces the data flow from individual nodes to the center node.

The results suggest that the radar with RadarNET is capable of estimating the position of the target even under heavy jamming. In the simulator, the average error of the proposed method is 50% smaller then using the classical approach as described in Ref. [7]. The reason for errors is due to the design of a jammer capable of confusing the correlation estimators. On the other hand, the neural network design using convolution layers can partially filter out the jamming signals, providing superior estimation accuracy. The most illustrative example of comparison with a moving target can be seen in Figure 13. The proposed RadarNET is able to follow the linear trajectory of the target.

In conclusion, the scripts used in this paper are readily available on GitLab [29] for further access and use. We encourage others to utilize these scripts in their studies and to contribute any modifications or improvements to the repository.

## Figures and Tables

**Table 1:** Table with errors for one jammer in the field of view. Our method using neural network processing is compared with the classical method for TDOA.

	Classical Method [m] [7]	Our Method [m]
Average position error	301.9	119.1
Maximal position error	1580	517.2

**Table 2:** Table with errors for two jammers transmitting Zadoff–Chu sequences. Our method, using neural network processing, is compared with the classical method for TDOA.

	Classical Method [m] [7]	Our Method [m]
Average position error	285.2	139.3
Maximal position error	2759	808.4

## Data Availability

The data with scripts are available on Gitlab [29].
